# Food-Derived Collagen Peptides, Prolyl-Hydroxyproline (Pro-Hyp), and Hydroxyprolyl-Glycine (Hyp-Gly) Enhance Growth of Primary Cultured Mouse Skin Fibroblast Using Fetal Bovine Serum Free from Hydroxyprolyl Peptide

**DOI:** 10.3390/ijms21010229

**Published:** 2019-12-28

**Authors:** Tomoko T. Asai, Fumi Oikawa, Kazunobu Yoshikawa, Naoki Inoue, Kenji Sato

**Affiliations:** 1 Division of Applied Biosciences, Graduate School of Agriculture, Kyoto University, Kyoto 606 8502, Japan; asai@cc.nara-wu.ac.jp (T.T.A.); kazunobu2017@gmail.com (K.Y.); 2 Department of Food Science and Nutrition, Faculty of Human Life and Environment, Nara Women’s University, Nara 630 8506, Japan; 3 Division of Applied Life Sciences, Graduate School of Life and Environment Sciences, Kyoto Prefectural University, Kyoto 606 8522, Japan; 23fumi.oioi@gmail.com; 4 Peptide Division, Nitta Gelatin Inc., 2-22 Futamata, Yao, Osaka 581 0024, Japan; na-inoue@nitta-gelatin.co.jp

**Keywords:** prolyl-hydroxyproline (Pro-Hyp), hydroxyprolyl-glycine (Hyp-Gly), collagen peptide, fibroblasts, fetal bovine serum (FBS)

## Abstract

Prolyl-hydroxyproline (Pro-Hyp) and hydroxyprolyl-glycine (Hyp-Gly) appear in human blood after ingestion of collagen hydrolysate and trigger growth of fibroblasts attached on collagen gel, which has been associated with beneficial effects upon ingestion of collagen hydrolysate, such as improvement of skin and joint conditions. In the present study, inconsistent results were obtained by using different lots of fetal bovine serum (FBS). Fibroblasts proliferated in collagen gel without adding Pro-Hyp and Hyp-Gly and did not respond to addition of Pro-Hyp and Hyp-Gly, which raises doubts about conclusions from prior research. Unexpectedly high levels of hydroxyprolyl peptides, including Pro-Hyp, however, were present in the FBS (approximately 100 µM), and also in other commercially available forms of FBS (70–80 µM). After removal of low molecular weight (LMW, < 6000 Da) compounds from the FBS by size exclusion chromatography, Pro-Hyp and Hyp-Gly again triggered growth of fibroblasts attached on collagen and increased the number of fibroblasts migrated from mouse skin. These results indicate the presence of bioactive hydroxyprolyl peptides in commercially available FBS, which can mask effects of Pro-Hyp and Hyp-Gly supplementation; our work confirms that Pro-Hyp and Hyp-Gly do play crucial roles in proliferation of fibroblasts.

## 1. Introduction

Collagen is the main protein in the extracellular matrix and has a triple-helical structure. Collagen has two specific post-translationally modified amino acids: hydroxyproline (Hyp) and hydroxylysine (Hyl). Heat treatment converts the triple-helical structure of collagen into a globular structure, which is referred to as gelatin. The protease digest of gelatin is referred to as collagen hydrolysate, gelatin hydrolysate, or collagen peptide. Collagen hydrolysate is prepared from skin, bones, and tendons of animals, or the skin and scales of fish. In human trials with placebo controls, ingestion of collagen hydrolysate (2.5–10 g/day) suppresses transepidermal water loss, reduces wrinkle volume, and increases elasticity of skin [1,2,3,4]. Furthermore, ingestion of collagen hydrolysate moderates the symptoms of osteoarthritis [5,6] and enhances healing of pressure ulcers [7,8,9]. Our previous work has shown that ingestion of collagen hydrolysate (2–20 g) increases the peptide forms of Hyp (i.e., hydroxyprolyl peptide or collagen peptide) in human peripheral blood plasma to 20–100 μM [10,11,12,13]. To date, the presence of more than ten food-derived hydroxyprolyl peptides in human blood has been reported, including prolyl-hydroxyproline (Pro-Hyp), hydroxyprolyl-glycine (Hyp-Gly), alanyl-hydroxyproline (Ala-Hyp), isoleucyl-hydroxyproline (Ile-Hyp), leucyl-hydroxyproline (Leu-Hyp), phenylalanyl-hydroxyproline (Phe-Hyp), glutamyl-hydroxyproline (Glu-Hyp), prolyl-hydroxyprolyl-glycine (Pro-Hyp-Gly), glycyl-prolyl-hydroxyproline (Gly-Pro-Hyp), alanyl-hydroxyprolyl-glycine (Ala-Hyp-Gly), and serinyl-hydroxyprolyl-glycine (Ser-Hyp-Gly). Pro-Hyp and Hyp-Gly are the main hydroxyprolyl peptides found in human blood after ingestion of collagen hydrolysate [10,11,12,13]. Pro-Hyp is also generated by the degradation of endogenous collagen in tissue undergoing inflammation [14] and at wound healing sites in the skin [15].

Mouse skin fibroblasts attached on collagen gel stopped growing without addition of Pro-Hyp, even in the presence of fetal bovine serum (FBS), whereas fibroblasts grew on plastic plates in the presence of FBS [11,16,17]. We previously reported that Pro-Hyp and Hyp-Gly triggered the growth of fibroblasts attached on collagen gel [11,17], which has been associated with biological responses upon ingestion of collagen hydrolysate. However, some researchers have obtained results inconsistent with our findings (personal communication). In the present study, we also found that fibroblasts attached on collagen gel grew without adding Pro-Hyp when we used different lots of FBS than those used in previous studies. The objectives of the present study were to solve this problem and confirm the effects of food-derived hydroxyprolyl peptides on the growth of fibroblasts.

## 2. Results and Discussion

### 2.1. Growth of Fibroblasts on Collagen Gel in Medium Containing FBS-1

**Figure 1:**
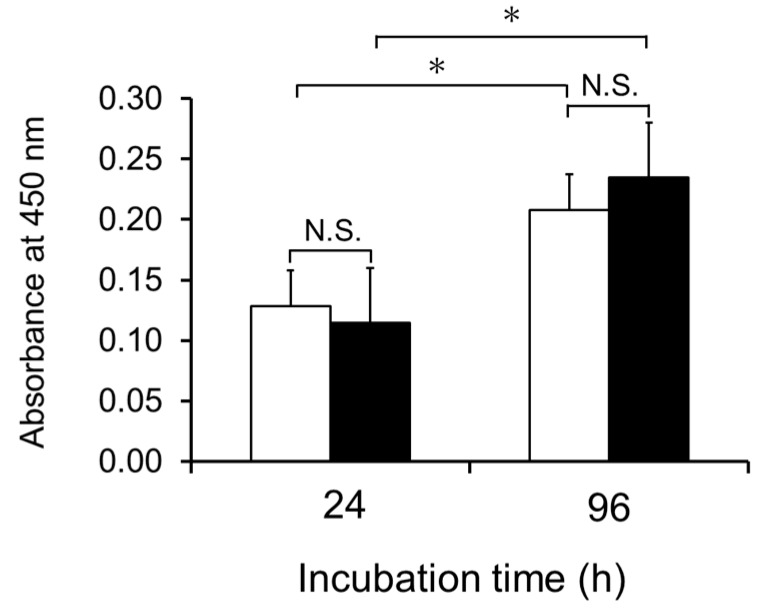
Effect of a mixture of Pro-Hyp and Hyp-Gly on the growth of fibroblasts on collagen gel in the presence of 10% FBS-1; (□), control; (■), medium containing Pro-Hyp and Hyp-Gly at 100 µM, respectively. Data are shown as mean ± standard deviation (SD) (*n* = 5). Asterisks indicate significant differences (*p* < 0.05; Tukey’s test). N.S. indicates results that are not significantly different.

Mouse skin fibroblasts were cultivated on collagen gel in a medium containing a commercially available lot of FBS (FBS-1). As shown in Figure 1, fibroblasts grew on the collagen gel even without addition of Pro-Hyp and Hyp-Gly. Addition of mixture of Pro-Hyp (100 μM) and Hyp-Gly (100 μM) did not significantly enhance growth of fibroblasts. These results are inconsistent with previous studies using different lots of the same brand of FBS [11,16,17], in which fibroblasts grew on collagen gel only after adding Pro-Hyp and Hyp-Gly. It has been demonstrated that some FBS lots contain significant amounts of free Hyp, while the presence of hydroxyprolyl peptide has not been examined [18]. We assumed that FBS might contain different levels of hydroxyprolyl peptides depending on lot number and brand, which might explain the inconsistent results.

### 2.2. Presence of Hydroxyprolyl Peptides in FBS

**Figure 2:**
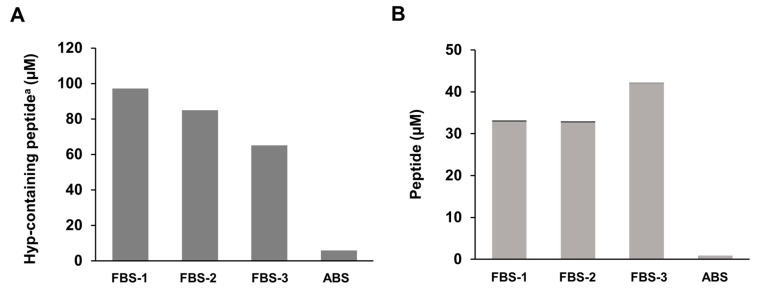
Contents of hydroxyprolyl peptide in commercially available fetal bovine serum (FBS) and adult bovine serum (ABS): (**A**) Hyp-containing peptide; (**B**) content of Pro-Hyp (■) and Hyp-Gly (■); ^a^ peptide form of Hyp.

Amino acid analysis revealed the presence of hydroxyprolyl peptides in the type of FBS used in the present study. As shown in Figure 2A, FBS contained unexpectedly higher levels of hydroxyprolyl peptide (approximately 70–100 µM) than adult bovine serum (ABS) and human plasma before ingestion of collagen hydrolysate [10,11,13]. As shown in Figure 2B, Pro-Hyp accounted for 37%–70% of total hydroxyprolyl peptides in FBS. These values are similar to those in human plasma after ingestion of collagen hydrolysate [10,11,13]. On the other hand, only negligible amounts of Hyp-Gly were present in the FBS. It has been demonstrated that constituents in FBS differ between lots, even from the same brand [18]. Thus, different lots of FBS might contain different levels of Pro-Hyp and other hydroxyprolyl peptides. 

### 2.3. Removal of Hydroxyprolyl Peptides from FBS-1

**Figure 3:**
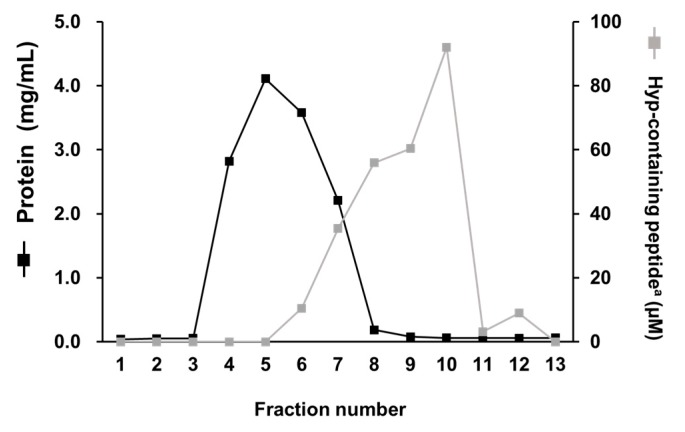
Elution of protein and hydroxyprolyl peptide in FBS-1 from the Econo-Pac 10DG column; (■), protein; (■), Hyp-containing peptide; ^a^ peptide form of Hyp.

As shown in Figure 3, the protein in FBS-1 was eluted in fractions 4–7 by size-exclusion chromatography (SEC). Hydroxyprolyl peptides were eluted in fractions 6–10. According to the instructions from the supplier, peptides larger than 6000 Da were eluted in fractions 4–7. Thus, hydroxyprolyl peptides in fractions 6 and 7 were larger than 6000 Da. Based on these facts, fractions 4–7 were collected and used as FBS-1 free from low molecular weight (LMW) compounds. Fractions 8–13 were collected and used as LMW fractions. 

**Figure 4:**
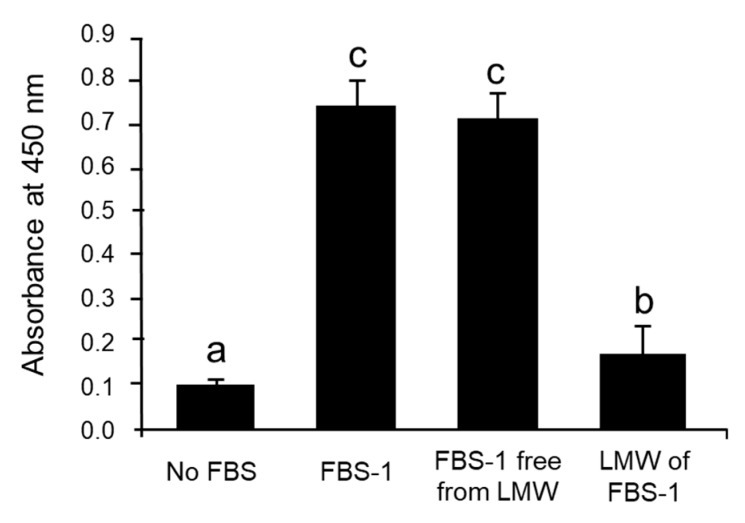
Effect of FBS-1 and its fractions on fibroblast growth on plastic plates. FBS-1 free from LMW, SEC Fr. 4–7; LMW of FBS-1, SEC Fr. 8–13. Data are shown as the mean ± SD (*n* = 5). Different letters indicate significant differences (*p* < 0.05, Tukey’s test).

The different fractions (FBS-1, FBS-1 free from LMW fractions, and LMW fractions) were each added to the medium of fibroblasts attached on plastic plates. FBS-1 free from LMW fractions caused fibroblast proliferation equivalent to proliferation with non-purified FBS-1 (Figure 4), while LMW fractions caused little fibroblast proliferation. These facts indicate that protein growth factors play a significant role in proliferating fibroblasts on plastic plates, as compared to LMW compounds in FBS.

### 2.4. Effect of Hydroxyprolyl Peptides on Growth of Fibroblasts on Collagen Gel

**Figure 5:**
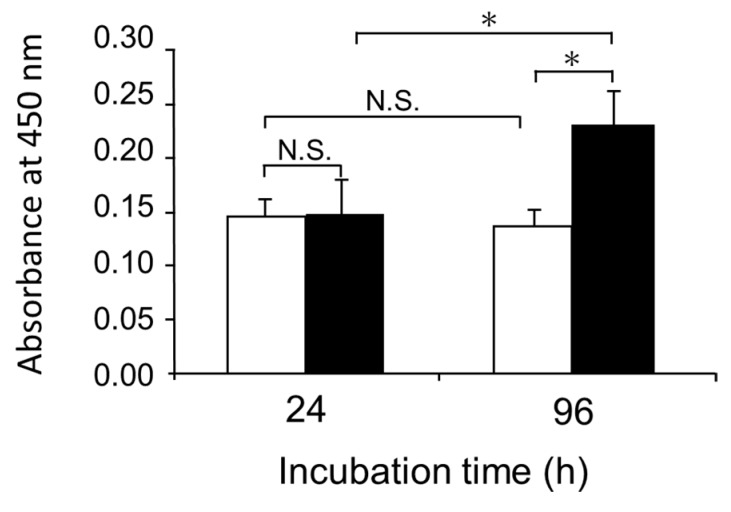
Effect of mixture of Pro-Hyp and Hyp-Gly on the growth of fibroblasts on collagen gel in the presence of 10% FBS-1 free from LMW hydroxyprolyl peptides; (□), control; (■), medium containing Pro-Hyp and Hyp-Gly at 100 µM. Data are shown as mean ± SD (*n* = 5). Asterisks indicate significant differences (*p* < 0.05, Tukey’s test). N.S. indicates results that are not significantly different.

As shown in Figure 5, fibroblasts attached on collagen gel stopped growing in the presence of FBS-1 free from LMW compounds. The addition of Pro-Hyp (100 μM) and Hyp-Gly (100 μM) triggered growth of the fibroblasts. These results are consistent with our previous studies [11,17]. However, when the present FBS-1 was used without purification, entirely different results were obtained (Figure 1) due to presence of hydroxyprolyl peptides in the FBS-1. 

### 2.5. Effect of Pro-Hyp on Number of Fibroblasts Migrated from Mouse Skin

**Figure 6:**
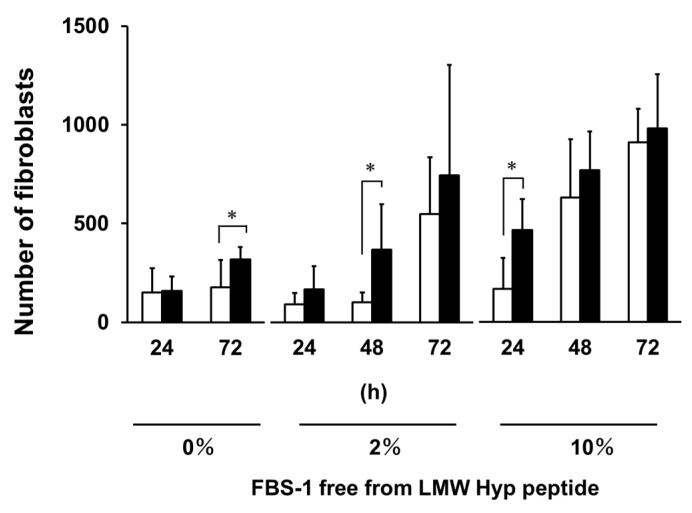
Effect of Pro-Hyp on the number of fibroblasts migrated from mouse skin in the absence or presence of FBS-1 free from LMW hydroxyprolyl peptides. Open column, control; closed column, medium containing Pro-Hyp at 200 µM. Data are shown as the mean ± SD (*n* = 6). Asterisks indicate significant differences (*p* < 0.05, Student’s *t*-test).

In our previous research, we demonstrated that Pro-Hyp (200 μM) increased the number of fibroblasts that migrated from skin in the absence of FBS [17]. However, the addition of FBS removed this effect [17]. The FBS-1 free from LMW compounds was added to the skin culture system to give 2% and 10% concentrations. As shown in Figure 6, the number of fibroblasts that migrated from skin was significantly increased by the addition of Pro-Hyp (200 μM) in absence of FBS after 72 h, which is consistent with previous results [17]. Even in the presence of FBS-1 free from LMW compounds at 2% and 10%, Pro-Hyp also increased the number of fibroblasts migrated from skin after 48 and 24 h, respectively. In the previous study [17], hydroxyprolyl peptides in FBS might have partially masked the effect of Pro-Hyp on the number of fibroblasts migrated from mouse skin. 

## 3. Materials and Methods 

### 3.1. Bovine Sera

Three different brands of FBS (FBS-1–3) were commercially obtained. One brand of ABS was commercially obtained. 

### 3.2. Chemicals

The amino acid standard mixture (Type H), acetonitrile (for high performance liquid chromatography: HPLC), trimethylamine, and phenyl isothiocyanate were all purchased from Wako Chemicals (Osaka, Japan). Hydroxyproline and 0.25% trypsin-ethylenediaminetetraacetic acid (EDTA) solution were purchased from Nacalai Tesque (Kyoto, Japan). Pro-Hyp and Hyp-Gly were obtained from Bachem (Bubendorf, Switzerland). Dulbecco’s phosphate-buffered saline (D-PBS) and gentamicin were purchased from Invitrogen (Carlsbad, CA, USA). Dulbecco’s modified Eagle medium (DMEM) supplemented with L-glutamine (584 mg/L) was from Sigma-Aldrich (St. Louis, MO, USA). Cell Counting Kit-8 was purchased from Dojin Glocal (Kumamoto, Japan). Calf acid-soluble type I collagen solution (0.5%; ⅠAC-50) was purchased from Koken (Tokyo, Japan). Here, 6-Aminoquinolyl-N-hydroxy succinimidyl carbamate (AccQ) was obtained from Waters Corporation (Milford, MA, USA). All other reagents were of analytical grade or better.

### 3.3. Removal of Hydroxyprolyl Peptides from FBS-1

An Econo-Pac 10DG column (Bio-Rad Laboratories, Hercules, CA, USA) was pre-equilibrated with the DMEM. In total, 3 mL of FBS-1 was loaded onto the column. After elution of the first 3 mL, 13 mL DMEM was loaded onto the column. Effluent was collected in 1 mL quantities. Elution of protein was monitored by the Bradford method using a Protein Assay Kit (Bio-Rad Laboratories). Hydroxyproline and hydroxyprolyl peptides were detected by amino acid analysis, as described previously [10].

### 3.4. Animals

All experiments were conducted according to the ethical guidelines of the Kyoto University Animal Research Committee. The protocol was approved by the Kyoto University Animal Research Committee (permission number: 2014–45, 2015–38). Five-week-old male Balb/c mice were purchased from Japan SLC (Shizuoka, Japan). Mice were sacrificed by cervical dislocation under deep isoflurane anesthesia. Abdominal skin was sterilized with 70% ethanol, shaved using a razor, and then stripped for use in these experiments.

### 3.5. Estimation of the Number of Cells Migrated from Mouse Skin

The skin was rinsed with D-PBS and DMEM to remove ethanol and placed on sterilized rubber plates. Disks measuring 4 mm in diameter were punched out using a Dermal Punch (Nipro, Tokyo, Japan). The skin disks were then placed on 12-well plastic plates (Falcon BD, Lakes, NJ, USA). DMEM was supplemented with gentamicin (0.01 mg/mL), FBS-1 (0%, 2%, and 10%) free from LMW hydroxyprolyl peptides, and Pro-Hyp (0 and 200 µM), respectively. Then, 1 mL of each mixture was added to the wells. The 12-well plastic plates were placed in a humidified incubator at 37 °C under 5% CO_2_. After incubation at suitable intervals, cells were fixed with 4% paraformaldehyde and observed using a phase-contrast microscope. The number of cells attached on the plate was directly counted.

### 3.6. Cell Proliferation Assay

Pieces of mouse abdominal skin (approximately 6–7 mm in width) were prepared using scissors and placed on a culture dish (90 mm i.d.). DMEM supplemented with gentamicin (0.01 mg/mL) and 10% FBS-1 (5 mL) were added into dish. The skin pieces were cultured in a humidified incubator at 37 °C under 5% CO_2_. During cultivation, the medium was changed every 2–3 days. After incubation for 2 weeks, the skin disks were removed; the fibroblasts migrated from skin were washed with PBS and treated with 1 mL of a 0.25% trypsin-EDTA solution at 37 °C for 10 min. Here, 9 mL of medium with 10% FBS-1 was poured into the plate to inactivate trypsin. Fibroblasts were collected by centrifugation at 3000 × *g* for 10 min. The pellets were suspended in each media: DMEM only, medium containing FBS-1, FBS-1 free from LMW hydroxyprolyl peptides, or LMW fraction of FBS-1 (5 × 10^4^ cells/mL), with or without the addition of Pro-Hyp (final concentration 100 µM) and Hyp-Gly (100 µM). Fibroblasts (5 × 10^3^ cells/100 µL) were cultured on 96-well plastic plates or collagen gel-coated plates in each media: DMEM only, medium containing FBS-1, FBS-1 free from LMW hydroxyprolyl peptides, or LMW fraction of FBS-1, with and without the addition of Pro-Hyp and Hyp-Gly. The collagen solution (0.5%) and the same volume of double-concentrated DMEM medium were mixed, and 100 µL of mixture was added into each well of the 96-well plastic plate. The plate was then incubated in a humidified incubator for 1 h at 37 °C under 5% CO_2_ to allow gelation. Cell proliferation was monitored using a Cell Counting Kit-8 instrument.

### 3.7. Amino Acid Analysis

FBS, ABS, and SEC fractions of FBS-1 were mixed with three volumes of ethanol and centrifuged at 1000 × *g* for 10 min. The supernatant was used as the 75% ethanol-soluble fraction. Next, 100 µL of the 75% ethanol-soluble fraction was dried in a glass tube (5 × 60 mm) and hydrolyzed by 6 M HCl vapor at 150 °C for 1 h, as described previously [10]. The Hyp contents in the non-hydrolysate and HCl hydrolysate were determined according to the method of Bidlingmeyer et al. [19], with slight modifications [10].

### 3.8. Determination of Pro-Hyp and Hyp-Gly

Pro-Hyp and Hyp-Gly in FBS were derivatized with AccQ and then determined by the liquid chromatography tandem mass spectrometry (LC-MS/MS) in multireaction monitoring (MRM) mode, using an LCMS-8040 (Shimadzu, Kyoto, Japan) and high-pressure binary gradient HPLC (LC20 system, Shimadzu), as previously reported [13].

### 3.9. Statistical analysis

Differences between means were evaluated using one-way analysis of variance, followed by Tukey’s multiple comparison test for post hoc analysis using GraphPad Prism Version 6.04 (GraphPad Software, San Diego, CA, USA). Differences between the two groups were compared using Student’s *t*-tests.

## 4. Conclusions

The present study demonstrates that some commercially available FBS contain high levels of LMW hydroxyprolyl peptides (70–100 μM), including Pro-Hyp. These values are higher than those in ABS and human plasma without ingestion of collagen hydrolysate. By using FBS that is free from LMW hydroxyprolyl peptides, the present study clearly confirms that Pro-Hyp and Hyp-Gly play crucial roles in proliferation of fibroblasts attached on collagen gel. It has been demonstrated that hydroxyprolyl peptides exert many functions, such as anti-hypertention [20], anti-inflammation [21], and improvement of glucose tolerance [22], in addition to improving skin and joint conditions. Therefore, researchers who use cell culture systems to evaluate biological activities should be aware of the presence of bioactive hydroxyprolyl peptides in FBS.

## Abbreviations

Pro-Hyp	prolyl-hydoxyproline
Hyp-Gly	hydroxyprolyl-glycine
Ala-Hyp	alanyl-hydroxyproline
Ile-Hyp	isoleucyl-hydroxyproline
Leu-Hyp	leucyl-hydroxyproline
Phe-Hyp	phenylalanyl-hydroxyproline
Glu-Hyp	glutamyl-hydroxyproline
Pro-Hyp-Gly	prolyl-hydroxyprolyl-glycine
Gly-Pro-Hyp	glycyl-prolyl-hydroxyproline
Ala-Hyp-Gly	alanyl-hydroxyprolyl-glycine
Ser-Hyp-Gly	serinyl-hydroxyprolyl-glycine
FBS	fetal bovine serum
LMW	low molecular weight
ABS	adult bovine serum
SEC	size-exclusion chromatography
HPLC	high performance liquid chromatography
EDTA	ethylenediaminetetraacetic acid
DMEM	Dulbecco’s Modified Eagle Medium
D-PBS	Dulbecco’s phosphate-buffered saline
AccQ	6-Aminoquinolyl-N-hydroxy succinimidyl carbamate
LC-MS/MS	liquid chromatography tandem mass spectrometry
MRM	multiple reaction monitoring
